# Cephalometric comparison of mandibular growth in Brazilian children with Class II malocclusion and normal occlusion during the growth stages determined by the cervical vertebrae maturation

**DOI:** 10.1590/2177-6709.28.5.e232358.oar

**Published:** 2023-11-03

**Authors:** Fabiane Louly Bapstista Santos SILVA, Ana Cláudia de Castro Ferreira CONTI, Thais Maria Freire FERNANDES, Priscila Pinto Brandão de ARAÚJO, Arnaldo PINZAN

**Affiliations:** 1 Universidade Anhanguera - Uniderp, Departamento de Ortodontia (Campo Grande/MS, Brazil).; 2 Private practice (Cuiabá/MT, Brazil).; 3 Universidade de São Paulo - USP, Faculdade de Odontologia, Departamento de Ortodontia (Bauru/SP, Brazil).

**Keywords:** Growth and development, Angle Class II, Cervical vertebrae

## Abstract

**Objective:**

This study compared mandibular growth in children, aged 7 to 12 years, with Class II malocclusion and normal occlusion, between the following stages of cervical vertebrae maturation: initiation (I), acceleration (A), transition (T).

**Material and Methods:**

A total of 148 lateral cephalograms (78 males, 70 females) of Class II malocclusion patients, and 60 lateral cephalograms (30 males, 30 females) of normal occlusion patients were included. The following linear cephalometric measurements were performed: Co-Gn (effective mandibular length), Co-Go (ramus height), and Go-Gn (length of mandibular body). Mean values of increments between stages (I-A, A-T, I-T) were obtained for each group and gender. Results were compared using the Student *t-*test, and a significance level of 0.05% was adopted.

**Results:**

Females group: A-T interval presented a greater increment in Co-Go in the Class II group, which was not significant for the I-T interval, with numerically smaller increments in Co-Gn and Go-Gn, without statistical significance. Males group: intervals I-A, A-T and I-T showed numerically smaller growth increments in the Class II group, with statistical significance for Co-Gn in I-A (*p*=0.001) and I-T (*p*=0.003). Comparing genders of the Class II group, Co-Go was higher in males (*p*=0.002) and I-T interval (*p*=0.031). In the Normal Occlusion group, the male gender had the greatest Co-Gn (*p*=0.038) for the I-A interval. In A-T and I-T, Co-Go in males was higher, with statistical significance (*p*=0.000 and *p*=0.002, respectively).

**Conclusion:**

Growth phenomenon affects the mandibular dimensions regardless of the character of the malocclusion, with a tendency to be smaller in the presence of Class II malocclusion.

## RESUMO

**Objetivo:**

Esse estudo comparou o crescimento mandibular em crianças de 7 a 12 anos de idade, com má oclusão de Classe II ou Oclusão Normal, entre os seguintes estágios de maturação das vértebras cervicais: iniciação (I), aceleração (A), transição (T).

**Material e Métodos:**

No total, 148 telerradiografias laterais (78 meninos, 70 meninas) de pacientes com má oclusão de Classe II e 60 telerradiografias laterais (30 meninos, 30 meninas) de pacientes com Oclusão Normal foram avaliadas. As medidas cefalométricas lineares Co-Gn (comprimento mandibular efetivo), Co-Go (altura do ramo mandibular) e Go-Gn (comprimento do corpo mandibular) foram analisadas e os valores médios dos incrementos entre os estágios (I-A, A-T e I-T) foram obtidos para cada grupo e sexo.

**Resultados:**

Os resultados foram comparados pelo teste *t* de Student, e o nível de significância adotado foi de 0,05%. Sexo feminino: houve maior incremento na altura do ramo no grupo Classe II em A-T, que diminuiu em I-T, com menores incrementos no comprimento mandibular efetivo e no corpo mandibular, sem significância estatística. Para o sexo masculino, nos intervalos I-A, A-T, I-T, os incrementos de crescimento foram numericamente menores no grupo Classe II, com significância estatística para o comprimento efetivo da mandíbula em I-A (*p*= 0,001) e em I-T (*p*= 0,003). No grupo Classe II, a altura do ramo foi maior para o sexo masculino (*p*= 0,002) e no intervalo I-T (*p*= 0,031). No grupo Oclusão Normal, o sexo masculino apresentou o maior comprimento mandibular efetivo (*p*= 0,038) no intervalo I-A. Nos intervalos A-T e I-T, a altura do ramo mandibular no sexo masculino foi maior e com significância estatística de *p*= 0,000 e *p*= 0,002, respectivamente.

**Conclusão:**

O fenômeno do crescimento afeta as dimensões mandibulares independentemente do característica da má oclusão, com tendência a ser menor na presença da má oclusão de Classe II.

## INTRODUCTION

Knowledge regarding the growth phenomenon is important in Orthodontics, especially for Class II malocclusion treatment, as the choice of a therapeutic approach to be used during maxillomandibular growth must be based on the patient’s skeletal maturation stage, since the success or failure of orthodontic treatment can be directly related to craniofacial growth and development.^1^

As Class II is the most frequent malocclusion in the orthodontist’s office,^2^ ^,^ ^3^ its particular characteristics are investigated in an attempt to identify the main cause responsible for the dysplasia, which can range from mandibular deficiency to maxillary protrusion,^4^ and thus, studies have reported that mandibular retrusion is the main cause of this malocclusion, with some exceptions.^5^

Some studies in the literature^6^ ^-^ ^10^ described the amount of annual growth increments that occur in children with normal occlusion, favoring the diagnosis and orthodontic treatment. However, few studies^8^ ^,^ ^11^ ^,^ ^12^ have evaluated craniofacial growth in Class II malocclusion by comparing it with a normal occlusion control group, thus demonstrating the growth alterations that occur in Class II in the pubertal growth stage, which is the ideal stage for an orthodontic treatment with better prognosis.

A systematic review concluded that orthodontic Class II treatment before the puberal growth spurt does not efficiently correct the skeletal problem, and the facial profile may not sufficiently improved.^13^ Thus, postponing treatment until onset of the growth spurt may result in a more favorable skeletal correction.^14^ ^-^ ^17^

The growth spurt period can be observed by assessment of hand-wrist radiographs or evaluation of cervical vertebrae maturation (CVM) stage.^18^ ^-^ ^20^ The CVM method has been used to predict when the maximum rate of facial growth occurs in Class II malocclusion.^12^ ^,^ ^20^ ^-^ ^22^ Some studies^19^ ^-^ ^22^ have correlated the period of greatest changes in cephalometric variables with the morphology of cervical vertebrae. However, these studies differed from the original method by Lampalski^18^ modified by Hassel and Farman^23^ and described by McNamara Jr and Franchi^24^ in details, based on the almost 20 years of experience in staging cervical vertebrae, in an article prepared as a “user’s guide”, in an attempt to help the professional using this approach in everyday clinical practice 

Thus, the aim of the present study was to evaluate the growth increments occurring in untreated mandibular Class II malocclusion using serial cephalograms, identifying the pubertal growth spurt between the stages of greater growth expectation (initiation, acceleration and transition), using the method by Lampalski^18^ modified by Hassel and Farman,^23^ described by McNamara Jr and Franchi.^24^

## MATERIAL AND METHODS

The retrospective experimental sample of this study belongs to the collection of the orthodontics department of the Bauru School of Dentistry at the University of São Paulo (São Paulo, Brazil). This study was designed as a cross-sectional research project. Group 1 consisted of 148 lateral cephalograms (78 males and 70 females), with a mean age of 10.03 years (minimum age 7; maximum age 12.87), belonging to the collection with skeletal Class II, division 1 malocclusion, based on their molar and canine relationships (Table 1); presenting ANB > 3°^12^ ^,^ ^25^, no previous orthodontic treatment; good quality lateral cephalograms, and NS.Ba angle within the range recommended by Bjork and Helm^25^ (between 125° and 132°). Group 2 consisted of 60 lateral cephalograms (30 males and 30 females), with an average age of 10 years (minimum age 6.69; maximum age 14.95), with normal occlusion, mild or absent crowding, without crossbite, with normal overjet and overbite, and no previous orthodontic treatment, belonging to the same collection of the discipline; good quality lateral cephalograms, with normal molar relationship, NS.Ba angle within the range recommended by Bjork and Helm^25^ (between 125° and 132°) (Table 1). 

**Table 1:** I-A, A-T and I-T intervals, for male and female genders in both groups.

CVM stages	Class II (n)	Normal occlusion (n)
Male		
I-A	65	22
A-T	54	22
I-T	37	16
Female		
I-A	59	27
A-T	53	22
I-T	28	11

n = number of patients. CVM = cervical vertebrae maturation (I = initiation, A = acceleration, T = transition).

**Figure 1:**
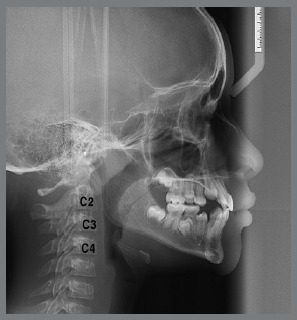
Initiation: lower edges of C2, C3 and C4 are flattened or flat, and upper edges are tapered from posterior to anterior. At this stage, pubertal growth and development is initiating, with an expectation of growth of 80% to 100%.

**Figure 2:**
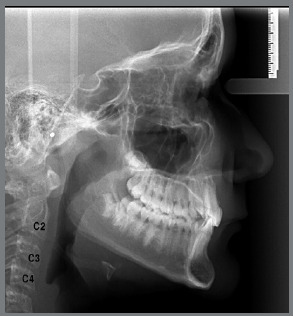
Acceleration: lower edges of C2 and C3 begin to show concavities, but the edge of C4 is still flat or flattened. The vertebral bodies of C3 and C4 are approximately rectangular in shape. At this stage, pubertal growth and development have already started, with an expectation of growth of 65% to 85%.

**Figure 3:**
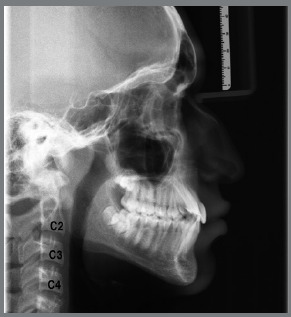
Transition: C2 and C3 present with distinct concavities on their lower borders, and C4 presents with onset of concavity on the lower border. The vertebral bodies of C3 and C4 are rectangular in shape. Pubertal growth is slowing down, but still has 25% to 65% of growth expectation.

The Class II group had a retrusive character of the mandible, in relation to the Normal Occlusion group (*p*=0.000). The power of the test was performed for the Co-Gn measure, considering the effect size equal to 1 and the significance level of 5%, with a mean for males and females of 84.5%. The ages of patients in each stage are shown in Table 2. All lateral cephalograms showed good quality, allowing the assessment of anatomical structures and cervical vertebrae (C2, C3 and C4), to score the skeletal maturation, using the method of Hassel and Farman,^23^ in: Initiation (I), Acceleration (A) and Transition (T) stages (Figs 1, 2 and 3).

**Table 2:** Descriptive statistics for ages (in years) in the CMV stages (*t*-test) , for both groups.

CVM stage	Female					Male					
	n	Mean	Minimum	Maximum	SD	n	Mean	Minimum	Maximum	SD	p
Class II											
I	17	8.09	7.00	9.6	0.83	24	8.49	7.07	10.52	0.88	0.148
A	43	9.99	8.03	12.16	1.07	41	10.45	8.57	12.59	0.93	0.040*
T	11	12.21	10.81	12.85	0.59	13	12.29	10.77	12.87	0.56	0.728
Normal occlusion											
I	8	7.51	6.90	8.05	0.49	8	7.85	6.69	8.76	0.65	0.275
A	19	10.08	8.77	11.98	0.89	14	10.49	9.27	12.00	0.89	0.203
T	3	12.40	11.68	13.05	0.68	8	12.70	11.05	14.95	1.14	0.676

* statistically significant at *p* < 0.05.

**Figure 4:**
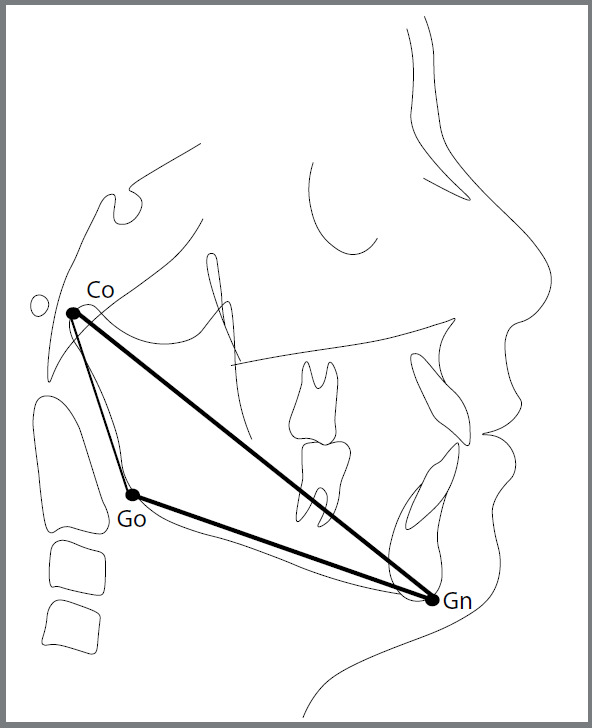
Lines and planes used in this study.

The cephalograms (Fig 4) were traced on Ultraphan transparent acetate paper, and the outlines of the second, third and fourth cervical vertebrae and the following anatomical structures were drawn: soft profile; middle anatomical porion; lower limit of the anterior margin of the foramen magnum, in the midsagittal plane; profile of frontal bone and nasal bones; Sela Turcica and body of the sphenoid; middle pterygomaxillary fissure; lower middle limit of the orbital cavities; maxilla; mandible (body and ramus); more prominent maxillary and mandibular central incisors; maxillary and mandibular first molars. The following points were marked: Co, Go and Gn, forming the variables Co-Gn (effective mandibular length), Co-Go (mandibular ramus height) and Go-Gn (mandibular body length), representing the mandibular size measures.

The sample consisted of three subgroups categorized according to CVM method.^24^ In this study, the sample was grouped according to three periods: prepubertal growth spurt (I-A), including subjects in the interval between CS1 and CS2 stages; pubertal growth spurt (A-T), including subjects in the interval between CS3 and CS4 stages; and I-T, to evaluate what happened between I and T phases.

The cephalograms were digitized using an Numonics AccuGrid A30TL digitizing table, connected to a microcomputer with a P3 700MHz Intel processor, to obtain the cephalometric measurements. The tracing and digitalization of points were performed by a single examiner, using the OrthoLateral pattern of the DentoFacial Planner 7.02 program, to obtain the measurements. Correction of the magnification factor (6% for the sample radiographs obtained up to 1995 and 9.8% for those obtained after 1996) was performed by the program.

## METHOD ERROR

To assess the intra-examiner error, forty-five lateral cephalograms were randomly selected,^26^ which were traced and digitized by the same researcher at an initial time (T1) and repeated after a 30-day interval (T2), according to Midtgard, Bjork and Linder-Aronson.^27^

To evaluate the systematic error as recommended by Houston,^28^ a paired *t-*test was used with a p-value <0.05. Application of the formula proposed by Dahlberg^29^ allowed estimating the order of random errors magnitude.

To assess the reliability of the CVM stages, data obtained at T1 and T2 were analyzed by Kappa agreement coefficient for intra-examiner evaluation, which, according to Landis and Koch,^30^ varies from -1 to 1.

The compatibility of groups regarding the proportion of genders, CVM stages, and ages was evaluated using the chi-square test.

## STATISTICAL ANALYSIS

The amount of increments between stages (I-A, A-T and I-T) in males and females was evaluated, thus obtaining the means and standard deviations for each variable, as well as the result of the *t-*test applied between the means, to identify statistically significant differences between the groups. Chi-square test was used to evaluated the compatibility of groups.

All statistical analyses were conducted using the Statistica software (Statistica for Windows, release 6.0, Copyright StatSoft, Inc), and results with *p*<0.05 were considered significant.

## RESULTS

No systematic errors were found, and random errors were considered acceptable, all of which were below 1mm for linear measurements (Co-Go=0.763mm; Co-Gn=0.604mm; Co-Gn=0.052mm). The Kappa coefficient was used to assess the intra-examiner agreement regarding the cervical vertebrae maturation stage (0.91=almost perfect), expressing high precision of the methodology adopted.

The compatibility of groups regarding the proportions of genders (*p*=0.559) and CVM stages (*p*=0.497), and for each CVM stage between groups [initiation stage (*p*=0.559); acceleration stage (*p*=0.497); transition stage (*p*=0.298)] was evaluated by the chi-square test, and demonstrated compatibility.

Detailed distributions of CVM stages for both sexes by chronological age are represented in Table 2, showing precocity of the stages in female patients.

The present study compared the mandibular growth between individuals with Class II and normal occlusion, using the first three CVM stages (considered the ones with higher growth expectations) of the Hassel and Farman^23^ method (modified by Lamparski and Nanda,^31^ and described by McNamara Jr and Franchi^24^).

The results of the comparison between the two groups for males and females at each CVM stage are shown in Table 3. In female gender, Co-Gn and Go-Gn were not significantly different between groups, while Co-Go was significantly different for Class II group. In the male group and stage I, the effective mandibular length (Co-Gn) and the mandibular ramus height (Co-Go) showed statistically significant differences between groups. Contrary to stage I, there was no significant difference for Co-Gn and Co-Go in stage A: male Normal Occlusion group showed a gain of 7.47mm in the effective mandibular length (Co-Gn), while the male Class II group obtained 3.63mm; the ramus height (Co-Go) in the male Normal Occlusion group had a gain of 3.14mm, while the male Class II group had only 1.68mm. For Go-Gn, the gain in the Normal Occlusion group was 4.84mm, while in the Class II group it was only 2.88mm. In stage T, the Class II male group presented an effective mandibular length (Co-Gn = 108.40mm) numerically smaller than that of Normal Occlusion group (Co-Gn = 109.73mm), but without significant differences (*p* = 0.488). This occurred due to the greater increment obtained in the male Normal Occlusion group (7.33mm) than in the Class II group (5.07mm). The Co-Go and Go-Gn measurements showed no difference between the groups, and greater increments were also observed in the Normal Occlusion group.

**Table 3:** Comparison between Class II and Normal Occlusion groups, for female and male genders, in the Initiation, Acceleration and Transition CVM stages, and *t-*test (p).

FEMALE															
Variable	INITIATION				*p*	ACCELERATION				*p*	TRANSITION				*p*
	Class II (n = 17)		Normal Occlusion (n = 8)			Class II (n = 42)		Normal Occlusion (n = 19)			Class II (n = 11)		Normal Occlusion (n = 3)		
	Mean	SD	Mean	SD		Mean	SD	Mean	SD		Mean	SD	Mean	SD	
Co-Gn	95.61	4.22	93.08	2.65	0.136	99.08	3.95	97.15	2.81	0.059	103.56	5.81	102.16	1	0.693
Co-Go	45.97	2.8	42.03	1.81	0.001*	47.53	2.67	44.02	2.36	0.000*	50.25	2.4	44.6	2.62	0.003*
Go-Gn	61.95	3.55	62.83	2.15	0.524	65.41	3.71	65.83	2.8	0.662	67.92	5.25	69.53	3.13	0.628
MALE															
Variable	INITIATION				*p*	ACCELERATION				*p*	TRANSITION				*p*
	Class II (n = 24)		Normal Occlusion (n = 8)			Class II (n = 41)		Normal Occlusion (n = 14)			Class II (n = 13)		Normal Occlusion (n = 8)		
	Mean	SD	Mean	SD		Mean	SD	Mean	SD		Mean	SD	Mean	SD	
Co-Gn	99.7	3.08	94.93	5.3	0.003*	103.33	3.26	102.4	7.23	0.511	108.4	3.13	109.73	5.59	0.488
Co-Go	46.02	2.78	43	2.88	0.013*	47.7	3.25	46.14	5.14	0.191	52.39	2.66	51.4	3.49	0.47
Go-Gn	65.53	3.23	62.57	4.72	0.055	68.41	3.77	67.41	4.43	0.416	69.98	3.35	69.75	5.03	0.898

*statistically significant at *p*<0.05.

To evaluate whether there was significant difference between the increments that occurred between stages (I-A, A-T, I-T), the amount of mandibular increments from Initiation to Acceleration (I-A), Acceleration to Transition (A-T) and Initiation to Transition (I-T) in females and males is shown in Table 4, with all variables representing mandibular dimensions being influenced by growth in the three studied stages.

**Table 4:** Growth increments in mandibular dimensions between CVM stages (I-A, A-T and I-T) for the female and male groups, and the comparison between groups (*t-*test).

FEMALE															
Variable	I-A				*p*	A-T				*p*	I-T				*p*
	Class II (n = 59)		Normal Occlusion (n = 27)			Class II (n = 53)		Normal Occlusion (n = 22)			Class II (n = 28)		Normal Occlusion (n = 11)		
	Mean	SD	Mean	SD		Mean	SD	Mean	SD		Mean	SD	Mean	SD	
Co-Gn	3.47	4.3	4.07	3.31	0.522	4.48	4.71	5.01	3.15	0.63	7.95	6.22	9.08	4.81	0.591
Co-Go	1.56	2.78	1.99	2.37	0.488	2.72	2.82	0.58	2.34	0.002*	4.28	3.36	2.57	2.26	0.129
Go-Gn	3.46	3.96	3	2.93	0.591	2.51	4.15	3.7	3.06	0.229	5.97	5.15	6.7	3.87	0.674
MALE															
Variable	I-A				*p*	A-T				*p*	I-T				*p*
	Class II (n = 65)		Normal Occlusion (n = 22)			Class II (n = 54)		Normal Occlusion (n = 22)			Class II (n = 37)		Normal Occlusion (n = 16)		
	Mean	SD	Mean	SD		Mean	SD	Mean	SD		Mean	SD	Mean	SD	
Co-Gn	3.63	3.63	7.47	7.43	0.001*	5.07	3.87	7.33	7.47	0.087	8.7	5.2	14.8	9.28	0.003*
Co-Go	1.68	3.17	3.14	4.64	0.102	4.69	3.7	5.26	5.21	0.591	6.37	4.1	8.4	5.32	0.137
Go-Gn	2.88	3.82	4.84	5.03	0.059	1.57	3.7	2.34	4.68	0.449	4.45	3.87	7.18	5.99	0.052

*statistically significant at *p* < 0.05. CVM = cervical vertebrae maturation (I = initiation, A = acceleration, T = transition).

Between female groups at I-A stage, the Class II group showed smaller amounts of growth increments in the variables that represent the mandibular dimensions; however, without significant difference. For the A-T stage, the amounts of growth increments in the effective length of the mandible (Co-Gn) and ramus body (Go-Gn) were smaller in the Class II group, still without significant difference; while the increment achieved in the mandibular height (Co-Go) was higher and statistically significant for the Class II group. For the I-T interval, growth increments occurred in the three studied variables, mainly in the Normal Occlusion group, but without significant difference, showing that the growth phenomenon occurred in all individuals in the studied stages, regardless of the malocclusion present. 

Between male groups for the I-A interval, the Class II group showed smaller amounts of growth increments in the variables that represent the ramus height (Co-Go) and the mandibular body length (Go-Gn), but without significant differences; while the effective mandibular length (Co-Gn) was statistically greater (*p*=0.001) in the Normal Occlusion group. For the A-T interval, all the increments that occurred in the mandibular variables remained numerically smaller in the Class II group, but without significant differences. For the I-T interval, the amount of growth increment in effective mandibular length (Co-Gn) was statistically smaller (*p*=0.003) in the Class II group, while in ramus height (Co-Go) and mandibular body length (Go-Gn), there were no statistically significant differences.

## DISCUSSION

Compatibility regarding gender ratio was tested between Class II and Normal Occlusion groups (Table 2), and, according to several studies, growth is more advanced in females.^9^ ^,^ ^32^

The maturation levels increase earlier in women,^33^ and indexes of maturation might provide a rough assessment of a patient’s current state of development.

The selected age ranges were wide enough to ensure including the growth peak in the sample, even tough maturation levels increase earlier in women,^9^ but only the Acceleration phase in the Class II group was statistically significant.

There was intergroup compatibility regarding the stages of skeletal maturation, ensuring the similarity between the growth stages between Class II and Normal Occlusion groups.

The Normal Occlusion sample in this study was not evaluated regarding growth type. A study on the effect of different growth types in Normal Occlusion subjects on the timing and rate of the mandibular growth peak found that the timing and rate of mandibular growth peak did not differ between vertical, average, and horizontal growth types.^9^ ^,^ ^34^

On the other hand, the Class II sample in this study presented a mean value for the mandibular plane angle (SN.GoGn=32.86°) between what Chung and Wong^8^ classified as a medium angle group (27° to 36°).

The aim in this study was to analyze mandibular length in subjects with Normal Occlusion and Class II skeletal patterns, considering their skeletal maturation stage. Then, this study focused in comparing mandibular growth increments, in children with Class II malocclusion and Normal Occlusion, from the maturational changes of the cervical vertebrae. Since the reproducibility and reliability of the CVM method was confirmed,^35^ and indicated that the intraobserver and interobserver agreements were substantial, it can be concluded that the current CVM classification method is reproducible and reliable. 

The increments of the mandibular dimensions are distributed over a wide range of chronological age intervals, which makes it unreliable to depend on chronological age to assess a patient’s skeletal growth stage.^35^

The Class II group (Table 4) showed smaller amounts of growth increments in the variables that represent the mandibular dimensions in I-A interval; however, without statistical significance. This is corroborated by Bishara et al^36^ and Bishara,^37^ who also found no significant difference in mandibular growth between individuals with Class II malocclusion and Normal Occlusion from primary to permanent dentition; and also by Stahl et al,^12^ except for Co-Gn in the normal occlusion male group, which was statistically significant (*p*=0.001) - the mean of this increment was between 3mm and 7mm.

At A-T interval, the increment in the mandibular height (Co-Go = 2.72mm) was higher and statistically significant for the Class II female group. This fact can be explained by the study of Chung and Wong,^8^ who stated that individuals with Class II who have a mean mandibular plane angle between 27° and 36° tend to have measurements of body length and ramus height greater than Class II individuals with SN.GoGn>36°. The Class II females sample in this study presented a value for the mandibular plane angle (SN.GoGn=32.86°) scored between what Chung and Wong^8^ classified as a medium angle group (27° to 36°), tending to present larger mandibular measurements. At I-T interval, growth increments occurred in the three studied variables, mainly in the Normal Occlusion group, but without statistical significance, showing that the growth phenomenon occurred in all individuals in the studied intervals, regardless of the malocclusion present. The increments were greater in the Normal Occlusion group, however, without statistical significance. 

In males group, the increments in Normal Occlusion group ranged from 4mm to 14mm, being higher in the Normal Occlusion group, and with statistical significance in I-A and I-T intervals, showing that the stages A and T are almost always involved with bigger increments, which is in accordance to Montasser,^9^ who also showed that the growth spurt peak almost always occurs either in the CVM2-CVM3 or the CVM3-CVM4 intervals. 

The mandibular growth deficiency in Class II subjects is significant not only in the growth spurt, but is also maintained at postpubertal observations.^12^ Although the current findings are based on cross-sectional data, the possibility that Class II dentoskeletal malocclusion does not have a tendency to self-correct with growth should not be neglected.^38^

## CONCLUSIONS

In females, there was a tendency for greater growth increments for the Normal Occlusion group at I-A interval. At A-T interval, Co-Go increment was significantly greater for the Class II group. At I-T interval, the increment values ​​tended to remain numerically higher for the Normal Occlusion group, but without significant differences.

The male gender showed numerically greater increments for all mandibular growth measurements in the Normal Occlusion group, with statistically significant differences for the effective mandibular length at I-A and I-T intervals.

Although the present study has evaluated CVM stages I to T in a 7 to 12 years old sample, it is suggested that future studies be carried out evaluating up to the last CVM stage, with a sample presenting a higher age range, to evaluate whether the phenomenon affects the mandibular dimensions regardless of the malocclusion character, with a tendency to be smaller in presence of Class II malocclusion in all CVM stages, and considering the possibility that Class II dentoskeletal malocclusion does not have a tendency to self-correct with growth.^38^
